# AIEE-Active Flavones as a Promising Tool for the Real-Time Tracking of Uptake and Distribution in Live Zebrafish

**DOI:** 10.3390/ijms241210183

**Published:** 2023-06-15

**Authors:** Yi Wu, Ying He, Huiqing Luo, Tingting Jin, Feng He

**Affiliations:** School of Pharmaceutical Science, Sun Yat-Sen University, Guangzhou 510006, China; wuyi76@mail2.sysu.edu.cn (Y.W.); heying67@mail2.sysu.edu.cn (Y.H.); luohq8@mail2.sysu.edu.cn (H.L.); jintt8@mail2.sysu.edu.cn (T.J.)

**Keywords:** AIEE, flavones, mitochondria, zebrafish, uptake, distribution

## Abstract

In recent years, aggregation-induced emission enhancement (AIEE) molecules have shown great potential for applications in the fields of bio-detection, imaging, optoelectronic devices, and chemical sensing. Based on our previous studies, we investigated the fluorescence properties of six flavonoids and confirmed that compounds **1**–**3** have good aggregation-induced emission enhancement (AIEE) properties through a series of spectroscopic experiments. Compounds with AIEE properties have addressed the limitation imposed by the aggregation-caused quenching (ACQ) of classic organic dyes owing to their strong fluorescence emission and high quantum yield. Based on their excellent fluorescence properties, we evaluated their performance in the cell and we found that they could label mitochondria specifically by comparing their Pearson correlation coefficients (R) with Mito Tracker Red and Lyso-Tracker Red. This suggests their future application in mitochondrial imaging. Furthermore, studies of uptake and distribution characterization in 48 hpf zebrafish larvae revealed their potential for monitoring real-time drug behavior. The uptake of compounds by larvae varies significantly across different time cycles (between uptake and utilization in the tissue). This observation has important implications for the development of visualization techniques for pharmacokinetic processes and can enable real-time feedback. More interestingly, according to the data presented, tested compounds aggregated in the liver and intestine of 168 hpf larvae. This finding suggests that they could potentially be used for monitoring and diagnosing liver and intestinal diseases.

## 1. Introduction

Organic fluorescent molecules have been gaining increasing popularity in imaging and sensing applications over the past decade for their ability to emit strong fluorescence [1,2]. This was demonstrated in a number of recent studies, showing that organic fluorescent molecules can be applied for use in cell and tissue sample imaging in the biomedical field [3]. However, their performance is highly restricted in terms of sensitivity, stability, and potency as they often show an aggregation-caused quenching (ACQ) problem, which limits their practical application in biological systems. When reaching a high local concentration in solution and forming aggregates, the fluorescein fluorophores are likely to suffer a strong π-π stack, inducing a sharp drop in fluorescence intensity in a process called “aggregation-caused quenching” (ACQ) [4,5,6,7]. Notably, the team of Tang reported novel fluorophores with aggregation-induced emission enhancement (AIEE) properties that bring the ultimate solution to this issue [8,9]. Opposite to conventional organic fluorescent molecules, the AIEE luminogens were found to be non-emissive or weakly luminescent materials in their solution state but became highly fluorescent at high concentrations or in a solid state, owing to the restriction of their intramolecular motions [10,11,12]. With more and more AIEE molecules being discovered [13,14,15], extensive work has been carried out. Different theories exist in the literature regarding the AIEE process, such as restricted intramolecular rotation (RIR), and J-aggregated and twisted intramolecular charge transfer (TICT). Our group has investigated the role of RIR in AIEE and has made considerable efforts to explain its mechanism [16]. Since the discovery of the AIEE-active materials’ unique fluorescent properties, they have been applied in ground-breaking applications, such as chemical/biological sensors, hazardous detection, optoelectronic devices, therapeutics, theranostics, and stimuli-responsive imaging studies [17,18,19,20,21,22,23]. The overall goal of our study is to identify a series of flavonoid compounds with AIEE properties and to explore their mitochondrial localization function as well as their real-time tracking ability in zebrafish.

Flavonoids are a class of polyphenolic compounds having a basic structural unit of 2-phenylchromone [24]. In recent years, flavonoids have attracted the attention of many researchers due to their remarkable array of biological and pharmacological effects as antioxidant, anti-inflammatory, antimicrobial, anticancer, antilipoperoxidant, anti-ischemic, and so on [25,26,27,28,29,30]. In our previous studies, we synthesized a series of flavonoids and flavanones and found that several of them had significant AIEE properties [31,32,33].

**Figure 1:**
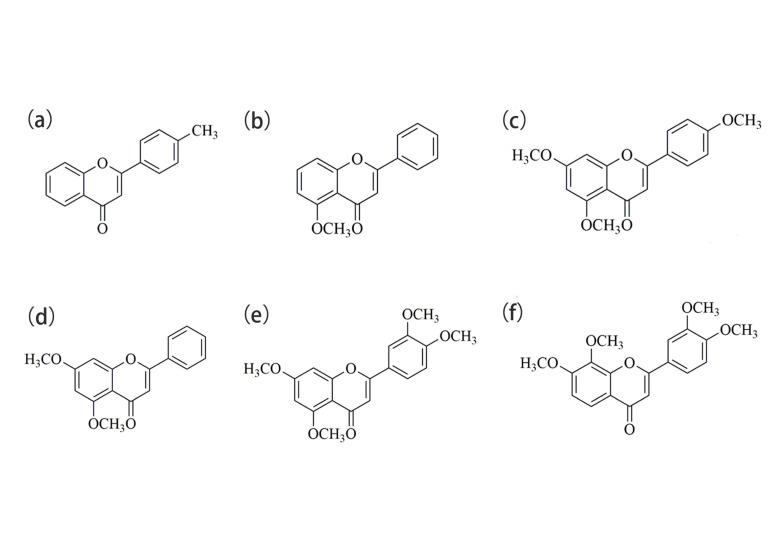
Chemical structures of compounds (**a**) **1**, (**b**) **2**, (**c**) **3**, (**d**) **4**, (**e**) **5**, and (**f**) **6**.

The overall goal of this paper was to explore the uptake and distribution capability in zebrafish of the six AIEE compounds of flavonoids (chemical structure is shown in Figure 1) screened by spectral experiments, together with their SEM and the A549 cell line imaging. Based on our previous work, we carried out several studies which have demonstrated that compounds **1**–**3** have significant AIEE properties and exhibit good fluorescence stability. Subsequently, through various viscosity tests, we speculated that RIR may be the main reason for the AIEE phenomenon of compounds **1**–**3**. Mitochondria are the driving force of the cell and affect key signaling pathways that are associated with homeostasis, proliferation, and cell death [34]. Fortunately, we found that compounds **1**–**3** not only labeled the mitochondria but also aggregated in the liver and intestine of zebrafish. The CCK-8 assay demonstrated that they have good biocompatibility and can specifically penetrate the cell membrane. Moreover, mitochondria can be specifically labeled by compounds **1**–**3**, which makes it easier to visualize the mitochondria. For further investigations, we also explored the uptake and distribution of these compounds on the 48 hpf larvae in the case of cycling different times (between uptake and utilization in the tissue) and explored the great potential of such AIEE compounds for zebrafish imaging and real-time drug visualization monitoring. Finally, we found that compounds **1**–**3** aggregated in liver and intestine of the 168 hpf larvae, which were fully developed at this time.

Recently, multiple studies have explored the close relationship between mitochondrial energy metabolism and liver dysfunction [35,36,37]. The liver is the busy site of mitochondrial energy metabolism [38]. Our findings during cell and zebrafish imaging indicated to us that compounds **1**–**3** may have potential for the visualization of mitochondria, and, secondarily, probably drug dynamics. The realization of the real-time monitoring of drugs in the body may help to pave the way for personalized diagnosis.

## 2. Results and Discussion

### 2.1. Optical Properties and Aggregation-Induced Emission Enhancement Properties

**Figure 2:**
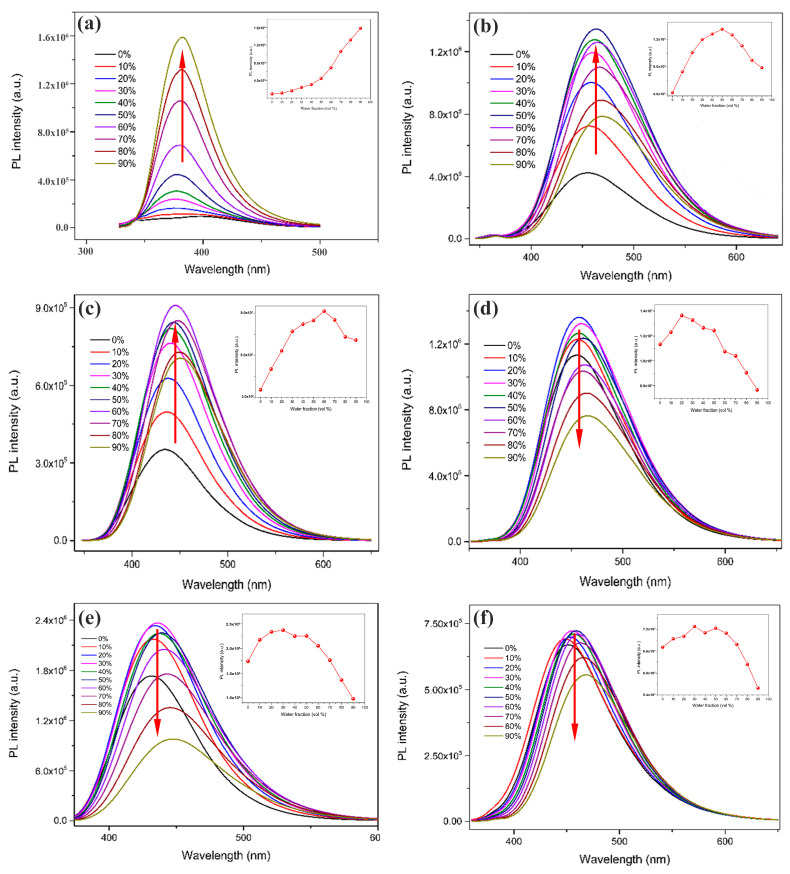
PL spectra of compounds (**a**) **1**, (**b**) **2**, (**c**) **3**, (**d**) **4**, (**e**) **5**, and (**f**) 6 in CH_3_OH/H_2_O mixed solutions (c = 2.07 × 10^−5^ M) with different water fractions (0–90%).

Compounds **1**–**6** are all easily soluble in methanol but not in water. To investigate their fluorescence properties, CH_3_OH/H_2_O mixed solutions containing 0–90% water were prepared and the emission values at room temperature were measured by PL spectroscopy. As shown in Figure 2a, the fluorescence intensity of compound **1** in the pure methanol solution was weak, but as the volume fraction of water in the CH_3_OH/H_2_O mixture solution gradually increased from 0% to 90%, the fluorescence intensity of the compound kept showing an increasing trend (we illustrated it with the upward-pointing red-arrow, b and c are the same as a) and reached the maximum value at the water content of 90%, at which time the maximum fluorescence intensity of the solution was about 17 times that of the maximum fluorescence intensity of the pure methanol solution, which indicated that compound **1** has obvious AIEE properties [39]. As shown in Figure 2b, the fluorescence intensity of the solution of compound **2** gradually increased with the increase from the pure water to the CH_3_OH/H_2_O mixture solution, and reached the maximum at 50% of water content. Subsequently, as the volume fraction of water in the mixed solution continued to increase to 90%, the fluorescence intensity showed a tendency to decrease probably due to a molecular aggregation mechanism [40]. This suggests that compound **2** is a molecule with AIEE properties. As can be seen in Figure 2c, the fluorescence intensity of the solution of compound **3** gradually increases with the increase in the volume fraction of water in the mixed solution and reaches a maximum at a water content of 60%. Then, when the water content in the mixed solution continues to increase, the fluorescence intensity of the solution slightly decreases. This indicates that compound **3** is a molecule with AIEE properties. Figure 2d shows the fluorescence spectra of compound **4** in CH_3_OH/H_2_O mixed solutions with different water contents. When the volume fraction of water in the mixed solution was increased from 0% to 20%, the fluorescence intensity was slightly enhanced; however, when the volume fraction of water continued to increase to 90%, the fluorescence intensity of the compound showed a significant decreasing trend (we illustrated it with the downward-pointing red-arrow, e and f are the same as d), which indicated that compound **4** had an ACQ property [41]. Figure 2e shows the fluorescence spectra of compound **5** in a CH_3_OH/H_2_O mixture solution with different water contents. When the water content of the mixed solution gradually increased from 0% to 30%, the fluorescence intensity of the solution slightly increased; however, when the water content continued to increase, the fluorescence intensity of the solution gradually decreased and reached the lowest value when the water content was 90%, which indicated that compound **5** was a compound with an ACQ property. Figure 2f shows the fluorescence spectra of compound **6** in the CH_3_OH/H_2_O mixture solution with different water contents. When the volume fraction of water in the mixed solution was increased from 0% to 30%, its fluorescence intensity increased slightly, but when the volume fraction of water continued to increase to 90%, the fluorescence intensity of the mixed solution gradually decreased and reached its lowest value at a water content of 90%, which indicated that compound **6** was a molecule with an ACQ property.

**Figure 3:**
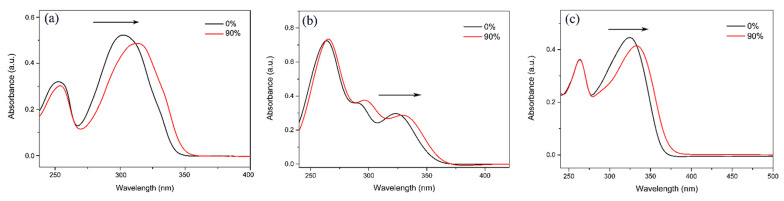
UV–vis absorption spectra of compounds (**a**) **1**, (**b**) **2**, and (**c**) **3** in pure CH_3_OH solution and CH_3_OH/H_2_O mixed solution with 90% water contents.

To further investigate the AIEE properties of compounds **1**–**3**, their UV–vis absorption spectra were measured in mixed solutions with 0% and 90% water content, respectively. The results are presented in Figure 3. In the pure methanol solution, the UV absorption wavelengths of compounds **1**–**3** were 302, 323, and 325 nm, respectively. When the water content in the CH_3_OH/H_2_O mixture solution increased to 90%, the UV absorption wavelengths of compounds **1**–**3** were all red-shifted (we used black-arrows to the right to illustrate it). This indicates that with the gradual Increase in the water content in the mixed solution, new aggregates were formed in the solution, which led to the gradual enhancement of the fluorescence emission of the compounds. This is consistent with our previous study [33].

**Figure 4:**
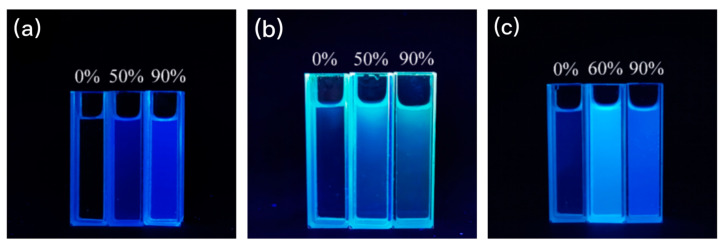
Fluorescence photographs of compounds (**a**) **1**, (**b**) **2**, and (**c**) **3** in CH_3_OH/H_2_O mixed solution (with various water fractions) under 365 nm wavelength UV light.

Figure 4a shows the fluorescence pictures of compound **1** with water contents of 0%, 50%, and 90% under 365 nm UV light. It can be observed that there is almost no fluorescence emission in the pure CH_3_OH solution, and when the volume fraction of water increases to 50%, the solution emits blue fluorescence; when the water content continues to increase to 90%, the fluorescence of the solution is obviously enhanced and a strong blue fluorescence can be observed. As observed in Figure 4b, when the water content was 0%, the solution emitted a very weak fluorescence; when the water content was 50%, a clear blue fluorescence could be captured; when the water content continued to increase to 90%, the intensity of the blue fluorescence emitted from the solution decreased, which was consistent with the trend of the fluorescence spectra. Similar results are observed in Figure 4c. This indicates that compounds **1**–**3** are molecules with AIEE properties.

To further investigate the AIEE properties of compounds **1**–**3**, we measured their fluorescence quantum yields (ϕF) in CH_3_OH/H_2_O mixed solutions with different water contents, respectively. As shown in Table 1, the ϕF of compound **1** in pure CH_3_OH solution was only 0.02; while when the water content increased to 50%, the ϕF increased to 0.16; when the water content continued to increase to 90%, the ϕF continued to increase and reached 0.29, and the trend was consistent with the fluorescence spectroscopy results. Compounds **2** and **3** were also observed to be consistent with the fluorescence spectra. These results further validate that compounds **1**–**3** have typical AIEE properties.

**Figure 5:**
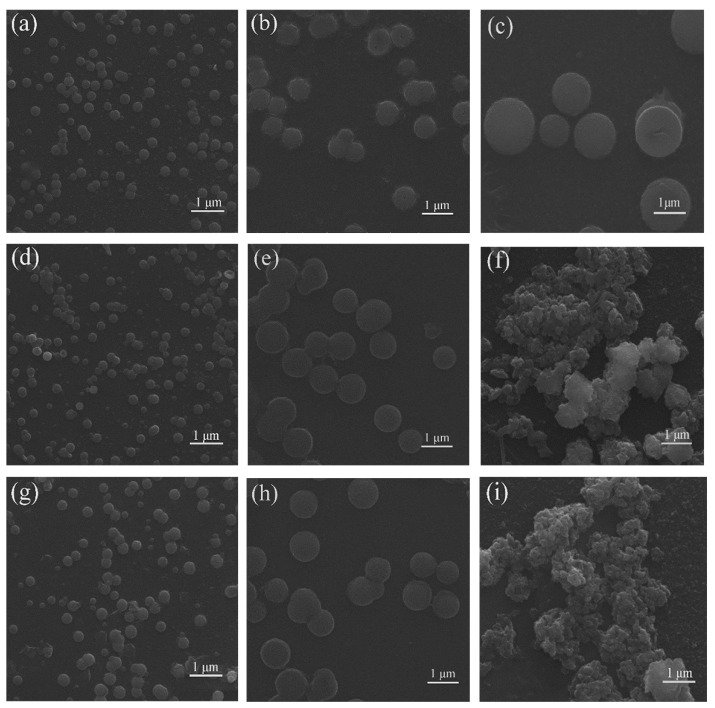
(**a**–**c**), (**d**–**f**) and (**g**–**i**) are SEM images of compound **1**, **2**, and **3**, respectively; and the first column is of CH_3_OH/H_2_O (5:5 *v*:*v*), the second (4:6 *v*:*v*), and the third (1:9 *v*:*v*).

In order to investigate the reason for the AIEE properties of compounds **1**–**3**, the morphology of compounds **1**–**3** in CH_3_OH/H_2_O mixed solutions with different water contents was tested by scanning electron microscopy (SEM) (Figure 5). Figure 5a–c show that the diameter of the spherical nanoparticles in solution increases with increasing water content. Figure 5d–i show the SEM images of compounds **2** and **3** in CH_3_OH/H_2_O mixed solutions with 50%, 60%, and 90% water content, respectively; when the water content is 50%, a large number of regular spherical nanoparticles with small diameters are distributed in the solution; when the water content increases to 60%, the diameters of spherical particles increase significantly, and spherical nanoparticles remain uniformly distributed in the solution; finally, when the water content continued to increase to 90%, larger irregular aggregates appeared in the solution, which may be the reason for the decrease in the fluorescence intensity of the compound at 90% water content [42]. The results indicate that the increase in the diameter of regular spherical nanoparticles is beneficial for the enhancement of fluorescence intensity, and when a large number of irregular nanoparticles are formed in the solution, the fluorescence emission is reduced to a certain extent.

**Figure 6:**
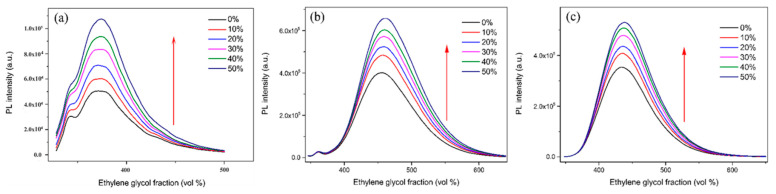
Fluorescence intensity of compounds (**a**) **1**, (**b**) **2**, and (**c**) **3** in CH_3_OH/EG mixed solutions with different EG fractions (0–50%).

In our previous study, we found that RIR is supposed to be the main reason for the AIEE properties of the flavonoids, flavones, and chalcones we investigated [32,33]. The same experimental approaches were used to explore the cause of the AIEE phenomenon of compounds **1**–**3**. The viscosity of the mixed solution was changed by adding different volume fractions of ethylene glycol, and then the fluorescence intensity of the compounds under different viscosity conditions was measured. The results are shown in Figure 6. When the volume fraction of EG in the CH_3_OH/EG mixed solution was gradually increased from 0% to 50%, the viscosity of the solution gradually increased, and the free rotation inside the molecule was restricted, and the fluorescence intensity of compounds **1**–**3** was gradually increased (we used the upward-pointing red-arrows to illustrate it). Based on the above results, we speculate that the main reason for the AIEE properties of compounds **1**–**3** may be the restricted intramolecular rotation (RIR), which is consistent with our previous study.

### 2.2. In Vitro Imaging of A549 Cells

**Figure 7:**
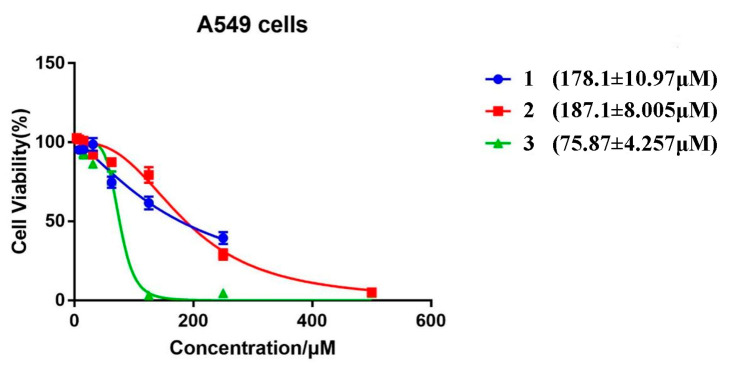
Cell viabilities of A549 cells co-incubated with different concentrations of compounds **1**–**3**.

The A549 cell lines are human lung cancer cells. The cytotoxicity of compounds **1**–**3** on A549 cells was determined through the CCK-8 assay to observe how compounds **1**–**3** behaved at the cellular level. The A549 cells were co-incubated with different concentrations of compounds **1**–**3** for 24 h, respectively, and the half-maximal inhibitory concentration (IC50) of each compound was quantified through GraphPad Prism 7.00 software, as shown in Figure 7 (178.1 ± 10.97, 187.1 ± 8.005 and 75.87 ± 4.257). The above results demonstrate that compounds **1**–**3** have low cytotoxicity and excellent cytocompatibility against A549 cells at the experimental concentrations for which they can be utilized as fluorescent visualizers for intracellular imaging.

**Figure 8:**
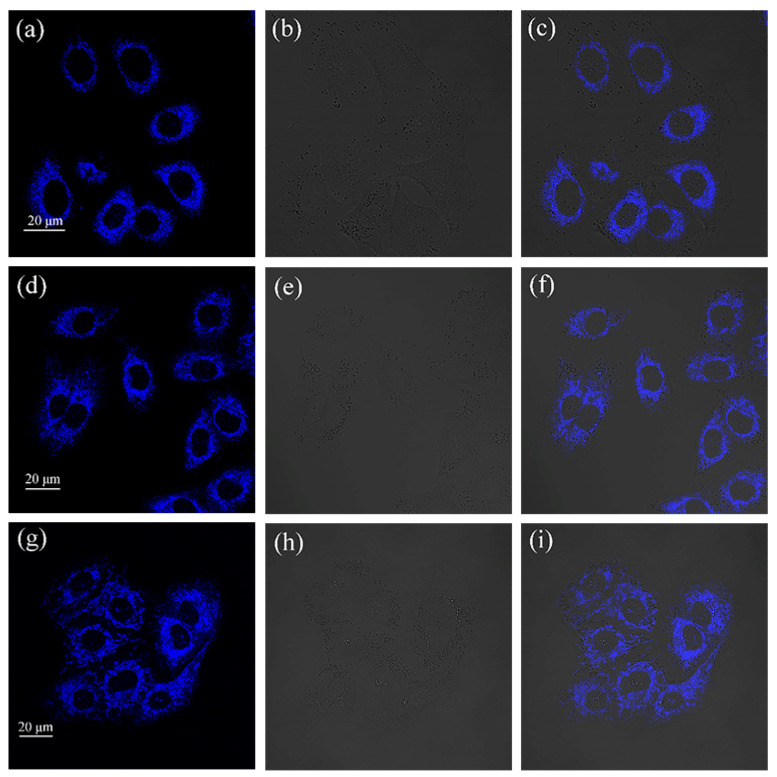
The imaging figures of A549 cells incubated with compounds (**a**) **1**, (**d**) **2**, and (**g**) **3** for 30 min with excitation at 405 nm. The bright-field images of A549 cells incubated with (**b**) **1**, (**e**) **2**, and (**h**) **3**. The merged images incubated with (**c**) **1**, (**f**) **2**, and (**i**) **3**.

**Figure 9:**
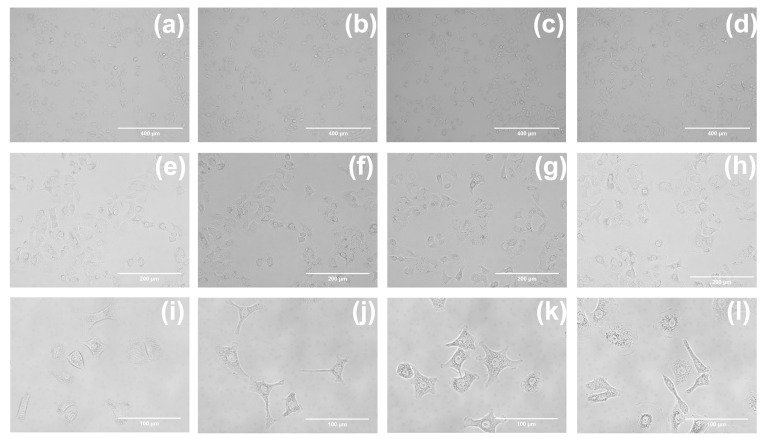
The morphological images of A549 cells. (**a**,**e**,**i**) The images of untreated control. The imaging figures of A549 cells incubated with compounds **1** (**b**,**f**,**j**), **2** (**c**,**g**,**k**), and **3** (**d**,**h**,**l**) for 24 h. (**a**–**d**) were taken with a fluorescence microscope under a 10-fold lens, (**e**–**h**) were taken under a 20-fold lens, and (**i**–**l**) were taken under a 40-fold lens. No difference in morphology was observed before or after incubating with compounds.

Cellular uptake of drug investigations were performed to further explore the application of compounds **1**–**3** in cell imaging. A549 cells were, respectively, incubated with of compounds **1**–**3** (20 μM) in a cell incubator for 30 min under dark conditions, washed with PBS three times, and finally observed by FV3000 laser scanning confocal microscopy. The intense blue fluorescence can be observed in the cytoplasm as shown in Figure 8. This indicates that compounds **1**–**3** can be taken up by A549 cells in a relatively short period of time and can aggregate in the cytoplasm, with good cytocompatibility and practical application possibilities for cell imaging. The morphological images of the untreated control and A549 cells incubated with compounds **1**–**3** for 24 h at different magnifications can be found in Figure 9. We added brightfield images at multiple fields of view in Figure S1. Brightfield images at multiple fields of view indicate that the compounds have no effect on cell morphology.

**Figure 10:**
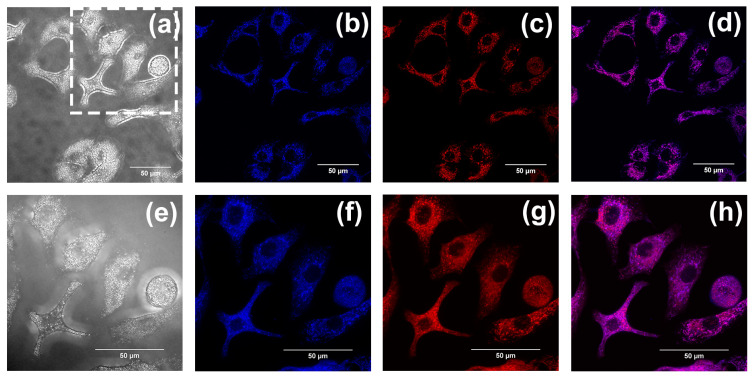
(**a**,**e**) The bright-field images of A549 cells incubated with compound **1**. Co-localized images of A549 cells incubated with compound **1** (20 µM) (**b**,**f**) and Mito Tracker Red (200 nM) (**c**,**g**) for 30 min. (**d**,**h**) The merged images co-cultured with compound **1**. The excitation wavelength of compound **1** was 405 nm. The excitation wavelength of Mito Tracker Red was 578 nm. (**a**–**d**) were taken with the confocal laser scanning microscope under a 60-fold lens, and (**e**–**h**) were taken under a 100-fold oil immersion lens. (**e**–**h**) are enlarged images of the dashed-square in (**a**). Scale bars, 50 µm.

**Figure 11:**
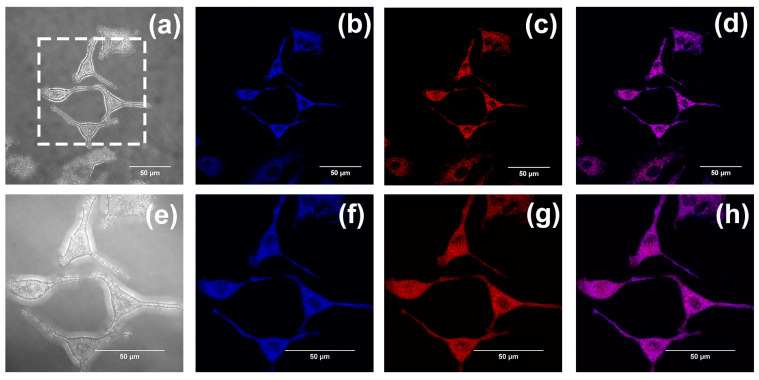
(**a**,**e**) The bright-field images of A549 cells incubated with compound **2**. Co-localized images of A549 cells incubated with compound **2** (20 µM) (**b**,**f**) and Mito Tracker Red (200 nM) (**c**,**g**) for 30 min. (**d**,**h**) The merged images co-cultured with compound **2**. The excitation wavelength of compound **2** was 405 nm. The excitation wavelength of Mito Tracker Red was 578 nm. (**a**–**d**) were taken using the confocal laser scanning microscope under a 60-fold lens, and (**e**–**h**) were taken under a 100-fold oil immersion lens. (**e**–**h**) are enlarged images of the dashed-square in (**a**). Scale bars, 50 µm.

**Figure 12:**
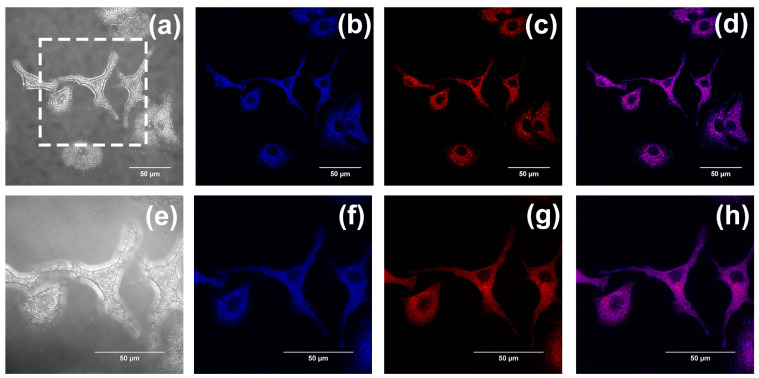
(**a**,**e**) The bright-field images of A549 cells incubated with compound **3**. Co-localized images of A549 cells incubated with compound **3** (20 µM) (**b**,**f**) and Mito Tracker Red (200 nM) (**c**,**g**) for 30 min. (**d**,**h**) The merged images co-cultured with compound **3**. The excitation wavelength of compound **3** was 405 nm. The excitation wavelength of Mito Tracker Red was 578 nm. (**a**–**d**) were taken using the confocal laser scanning microscope under a 60-fold lens, and (**e**–**h**) were taken under a 100-fold oil immersion lens. (**e**–**h**) are enlarged images of the dashed-square in (**a**). Scale bars, 50 µm.

**Figure 13:**
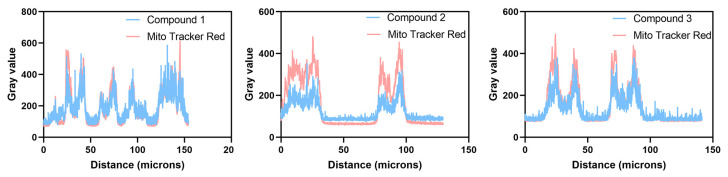
The Plot Profile of compounds **1**–**3**.

According to our previous studies, flavonoids, flavanones, and chalcones are specifically aggregated in mitochondria [32,33]. Mitochondrial co-localization experiments were also conducted to investigate whether compounds **1**–**3** could aggregate in mitochondria. The A549 cells were separately cultured with Mito Tracker Red (MT) (200 nM) and compounds **1**–**3** (20 µM) and were washed with PBS three times after incubation for 30 min. Last, cell imaging was performed with the confocal laser scanning microscope, using both a 60-fold and 100-fold oil immersion lens. Fluorescence images were recorded at an interval of 1.0 μm in the longitudinal (Z) direction. Images of cells after being stained with compounds **1**–**3** are shown in (b,f) in Figure 10, Figure 11 and Figure 12, with the cytoplasm emitting a clear blue fluorescence. Images of cells stained with Mito Tracker Red are shown in (c,g) in Figure 10, Figure 11 and Figure 12. Mito Tracker Red allows for the specific visualization of mitochondria and emits an intense red fluorescence. An observation of the merged images (d,h) in Figure 10, Figure 11 and Figure 12 reveals that the blue fluorescent region stained with the compounds and the red fluorescent region, specifically labeled by Mito Tracker Red, are highly overlapping. To precisely analyze the coincidence, we performed a quantitative analysis through the Fiji Java 1.8.0_322 software, and the results are shown in Table 2 below. The Pearson correlation coefficients (R, range −1 to +1) of compounds **1**–**3** after co-staining with the Mito Tracker Red were 0.86, 0.87, and 0.85, respectively, which indicated that compounds **1**–**3** could specifically aggregate in the mitochondria. The gray value of the Plot Profile (Figure 13) also supported this. We added the two-dimension co-localized images of A549 cells incubated with compounds **1**–**3** (20 µM) and Mito Tracker Red (200 nM) for 30 min in Figure S11. Co-localized images of A549 cells incubated with our compounds (20 µM) and Lyso-Tracker Red (200 nM) for 30 min can be found in Figures S2–S5 and S12, and Table S1 in the Supplementary Materials to further support our results.

### 2.3. Real-Time Tracking in Zebrafish

**Figure 14:**
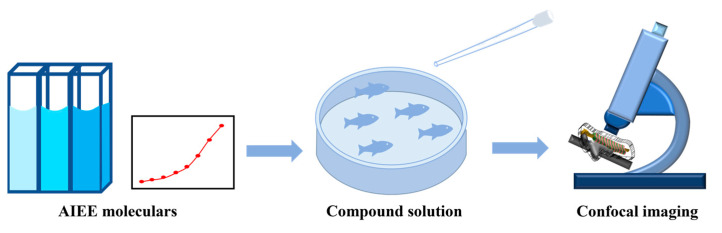
Experimental procedure.

**Figure 15:**
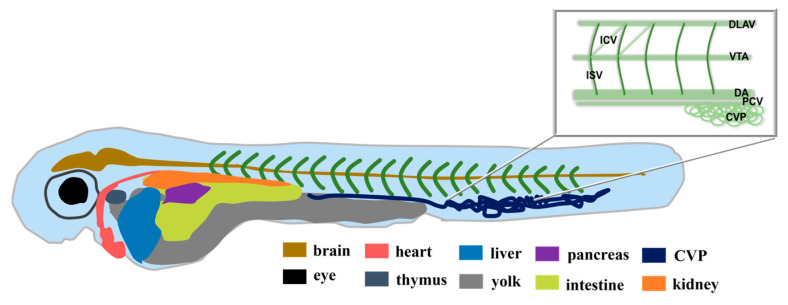
Structural sketch of zebrafish. DLAV, dorsal longitudinal anastomotic vessels; ICV, intercostal vessels; VTA, vertebral arteries; ISV, intersegmental vessels; DA, dorsal aorta; PCV, posterior cardinal vein; CVP, caudal vein capillary plexus.

The above results indicate that compounds **1**–**3** are viable for cellular imaging. Zebrafish were used as our experimental subjects to further investigate the application of compounds **1**–**3** in bioimaging. Their main advantages over other animal models include their small size, relative ease of reproduction and maintenance under laboratory conditions, relatively short generation time (3 months), and high fecundity. In addition, embryos and larvae are transparent, allowing for imaging of the development and physiological functions of many internal tissues and organs [43,44]. The experimental procedure is briefly depicted in Figure 14. Figure 15 is a structural sketch of zebrafish. We have marked the main organs of zebrafish in the figure and introduced the zebrafish blood vessels in the upper right corner. Compound **2** was selected and is discussed in the following paragraph, and the consistent results of compound **1** and **3** can be seen in the Supplementary Materials (Figures S6 and S7).

**Figure 16:**
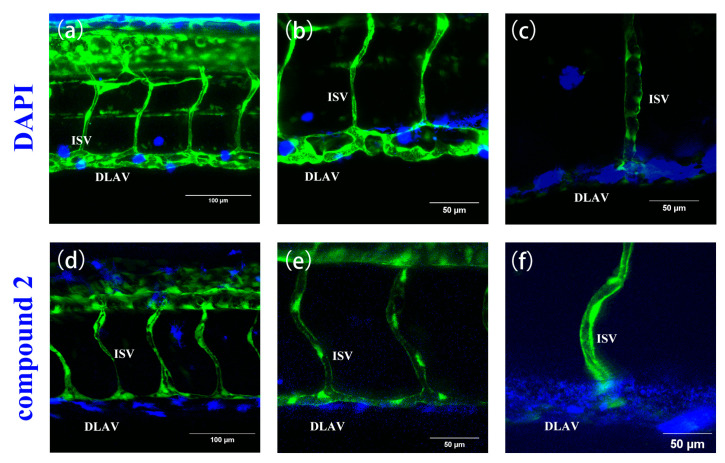
The fluorescence images of Tg(fli1a:EGFP) after incubation, respectively, with DAPI (10 µg/mL) (**a**–**c**) and compound **2** (40 µM) (**d**–**f**) for 1 h at 48 hpf. Compound **2** and DAPI exhibit similar permeability and both emit strong blue fluorescence. Zebrafish blood vessels are characterized by green fluorescence. Scale bars, 100 µm (**a**,**d**), 50 µm (**b**,**c**,**e**,**f**).

**Figure 17:**
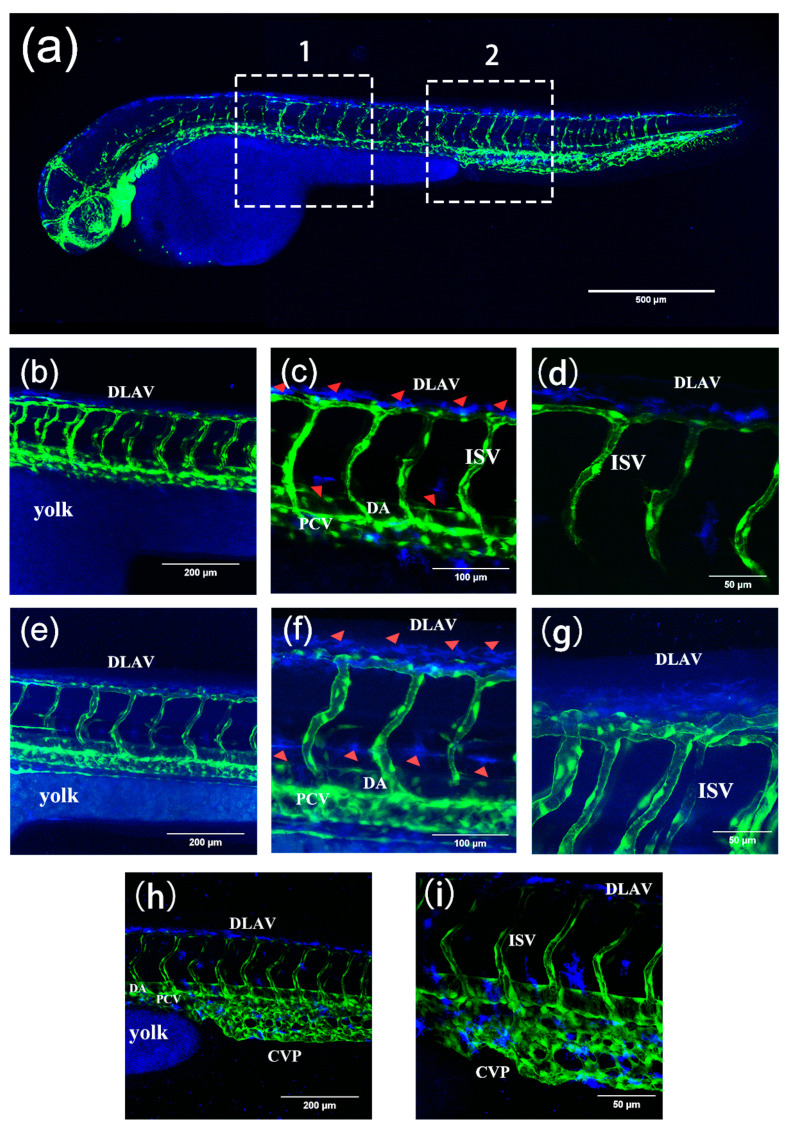
(**a**) The 3D images of zebrafish trunk vessels. (**b**–**g**) and (**h**,**i**) are enlarged images of areas 1 and 2 in (**a**), respectively. (**b**–**d**) Fluorescence images of Tg(fli1a:EGFP) after incubation with compound **2** (40 µM) for 1 h in 48 hpf larvae. In (**c**), most of the compounds adhere to the surface of the blood vessels, and a few enter the blood vessels. (**e**–**g**) Fluorescence images of Tg(fli1a:EGFP) after incubation with compound **2** (40 µM) for 2 h at 48 hpf. Arrowheads in (**c**,**f**) indicate that compound **2** adheres to the surface of the blood vessels. In (**f**), the amount of compound absorbed into the blood vessels is greatly increased. In (**h**,**i**), compound **2** enters the CVP. Zebrafish blood vessels are characterized by green fluorescence. Scale bars, 500 µm (**a**), 200 µm (**b**,**e**,**h**), 100 µm (**c**,**f**), and 50 µm (**d**,**g**,**i**).

**Figure 18:**
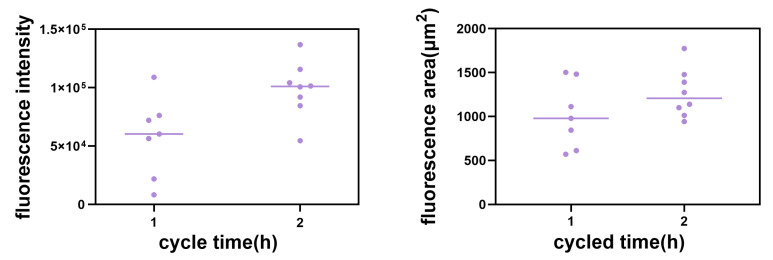
Fluorescence intensity of compound **2** cycled for 1 h and 2 h, respectively, and fluorescence area of compound **2** cycled for 1 h and 2 h, respectively. The number of dots in Figure 18 corresponds to the red triangles in Figure 17.

**Figure 19:**
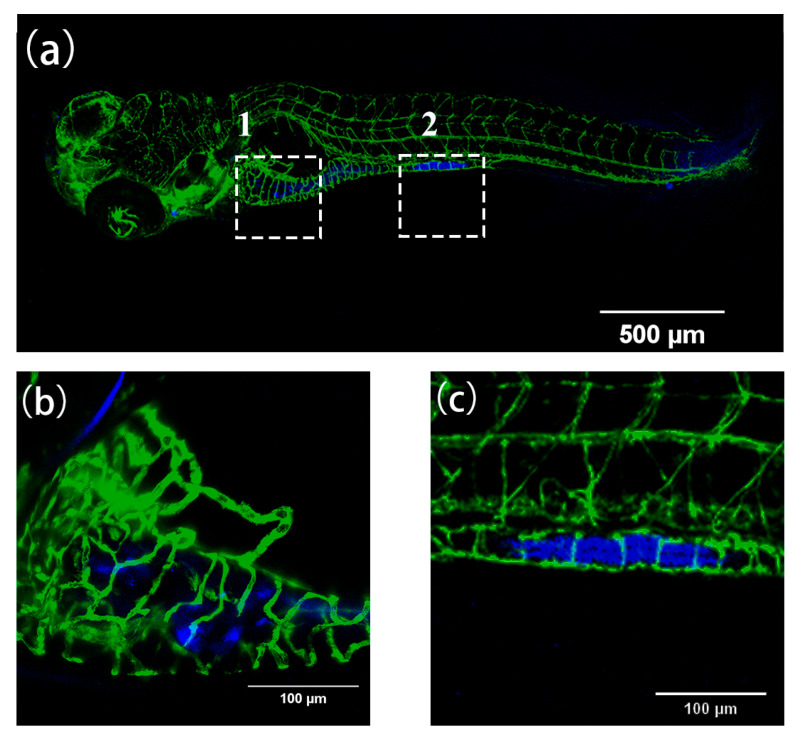
(**a**) The fluorescence images of Tg(fli1a:EGFP) after incubation with compound **2** (40 µM) for 2 h at 168 hpf. (**b**) and (**c**) are enlarged images of areas 1 and 2 in (**a**), respectively. Zebrafish blood vessels are characterized by green fluorescence. Scale bars, 500 µm (**a**), 100 µm (**b**,**c**).

Permeability experiments with compound **2** showed that it was able to enter 48 hpf (where the major blood vessels of body are developed) larvae rapidly within an hour, emitting strong blue fluorescence at 40 μM, similar to the permeability of the classical dye DAPI at 10 μg/mL (Figure 16a–f). Therefore, we have tried to alter the time between uptake and utilization in the tissue of compound **2** in 48 hpf zebrafish to further investigate the uptake, which means using the same incubation time but a different imaging time. We found that there were obvious changes between different cycle times regarding the concentration of compound **2** near the trunk vessel (a series of major blood vessels in the zebrafish’s body). It can be seen that zebrafish cycled with compound **2** (40 μM) for 2 h (Figure 17e–g) showed a significant fluorescence area (red triangles indicate compounds) larger than 1 h (Figure 17b–d and Figure 18). Moreover, we also found that compound **2** could enter the caudal venous plexus (CVP) (Figure 17h,i). Another promising finding was that compound **2** (40 μM) could concentrate in liver and intestine of 168 hpf (fully developed embryos, whose blood vessels of the liver and intestine can be visualized) larvae after cycling for 2 h (Figure 19b,c). It can be seen that the intense blue fluorescence is concentrated in the liver and intestine, while there is little distribution of the drug in the trunk vessels. We speculate that this may be due to changes in the tissue of the skin that form some sort of barrier at 168 hpf [45]. All the above findings indicate that our AIEE compounds can be visualized in real time in vivo and may help to monitor the diseases of the liver and intestine.

## 3. Materials and Methods

### 3.1. Materials and Instruments

#### 3.1.1. Chemical Compounds Used

All the regents and analytical grade solvents were obtained commercially and used without further purification unless indicated. O-hydroxyacetophenone, 4-methylbenzoyl chloride, pyridine, potassium hydroxide(KOH), hydrochloric acid, glacial acetic acid, concentrated sulfuric acid, methanol, petroleum ether, ethyl acetate, 5-Methoxyflavone (compound **2**), 4′,5,7-Trimethoxyflavone (compound **3**), 5,7-Dimethoxyflavone (compound **4**), 3′,4′,5,7-Tetramethoxyflavone (compound **5**), and 3′,4′,7,8-Tetramethoxyflavone (compound **6**) were purchased from Macklin, Sigma-Aldrich, and Aladdin (Shanghai, China).

#### 3.1.2. Cell

The human lung cancer cell line A549 was purchased from the Wuhan Procell Biological Technology Co. (Wuhan, China). The cell line was authenticated and confirmed negative for mycoplasma contamination by the provider. Dulbecco’s modified Eagle’s Medium (DMEM), 0.25% trypsin solution, phosphate-buffered saline (PBS), Cell Counting Kit-8 (CCK8), and Mitotracker Red assay kit (MT) were obtained from Thermo-Fisher Biochemical Products (Beijing, China).

#### 3.1.3. Zebrafish

All zebrafish were kept at a constant temperature of 28.5 °C and were on a 14 h light-on/10 h light-off cycle. At 4 h post-fertilization (hpf), we removed the abnormally developed and dead embryos. All the zebrafish embryos that developed beyond 24 hpf were treated with a culture containing PTU(0.003%, *w*/*v*; Sigma, Shanghai, China). The Tg(fli1a:EGFP)^y1^ line was available through the Zebrafish Resource Center (Core Lab Plat for Medical Science, Zhongshan School of Medicine, Sun Yat-sen University). We used 48 hpf (for whom the major blood vessels of body are developed) and 168 hpf (for whom the blood vessels of the liver and intestine can be visualized) embryos to conduct experiments. All procedures were conducted in accordance with the Code of Ethics for Animal Experiments of Sun Yat-sen University. The ethical approval number is SYSU-IACUC-2020-B0659.

#### 3.1.4. Scientific Instruments

The ^1^H-NMR (400 MHz) and ^13^C-NMR (101 MHz) spectra were obtained on an Avance III 400 MHz spectrometer (Bruker, Karlsruhe, Germany) in CDCl_3_. The progress of the reaction was checked by analytical thin-layer chromatography (TLC). The mass spectra were measured using an LCMS-IT-TOF mass spectrometer (Shimadzu, kyoto, Japan). Photoluminescence (PL) spectra and absolute PL quantum yields were obtained via an FLS920 spectrophotometer (Edinburgh Instruments, Edinburgh, UK). Ultraviolet (UV) absorption spectra were obtained using a UV-2600 spectrometer (Shimadzu, Kyoto, Japan). Scanning electron microscope (SEM) images were obtained using a Zeiss Merlin emission scanning electron microscope (Zeiss Co., Oberkochen, Germany). Brightfield pictures were taken with a fluorescence microscope (EVOS fl AMG; Westover Scientific, Bothell, WA, USA). Fluorescent images were examined on an OLYMPUS FV3000 laser scanning confocal microscope (Zeiss Co., Oberkochen, Germany), and cell viabilities were analyzed using a Flex Station 3 microplate reader (Molecular Devices, Silicon Valley, San Jose, CA, USA).

### 3.2. Synthesis of 4′-Methyflavanone

**Figure 20:**
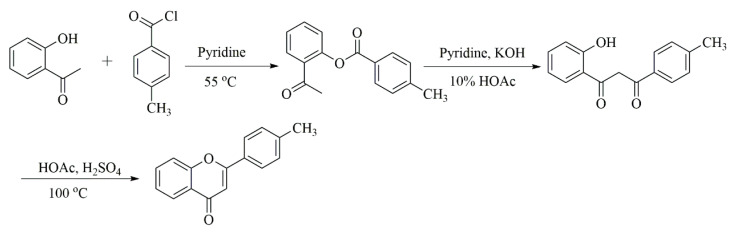
Synthetic route and chemical structures of 4′-methyflavanone.

The general synthesis procedure of 4′-methyflavanone is exhibited in Figure 20. This synthesis requires three steps. The first step is the Schotten–Baumann reaction between O-hydroxyacetophenone and 4-methylbenzoyl chloride, catalyzed by pyridine to produce O-acetylphenol p-toluate. The second step is the Baker–Venkataraman rearrangement reaction of O-acetylphenol p-toluate in the presence of potassium hydroxide to produce the corresponding propanedione. Finally, propanedione was cyclized under the co-catalysis of glacial acetic acid and concentrated sulfuric acid to produce the final target product 4′-methyflavanone [46,47]. Fortunately, the final product was obtained in good yield. The characterization of compound **1** can be seen in the Supplementary Materials Figures S8–S10.

#### 3.2.1. Synthesis of Compound **1**

O-hydroxyacetophenone (0.9 mL), 4-methylbenzoyl chloride (1.1 mL), and dry distilled pyridine (2.5 mL) were added to a 50 mL round-bottom flask equipped with a reflux condenser and stirred for 30 min at 55 °C in a constant temperature oil bath. Then, the mixture was poured into hydrochloric acid (60 mL, 1 mol/L) mixed with 25 g of crushed ice and stirred continuously until a solid was formed. It was extracted and then washed with 2.5 mL ice/methanol and 2.5 mL water, respectively. Finally, it was recrystallized from methanol/water. After suction filtration and drying, O-acetylphenol p-toluate was obtained.

O-acetylphenol p-toluate (2.5 g), potassium hydroxide powder (0.85 g), and dry distilled pyridine (9 mL) were added to a 100 mL round-bottom flask equipped with a reflux condenser and stirred for 20 min at 55 °C in a constant temperature oil bath. After the flask was cooled at room temperature for 10 min, 10% acetic acid solution (12.5 mL) was added and stirred continuously until a solid was formed, and 1-(2-Hydroxyphenyl)-3-(4-methylphenyl)-1,3-propanedione was obtained after filtration, washing, and drying.

1-(2-Hydroxyphenyl)-3-(4-methylphenyl)-1,3-propanedione (2.0 g), glacial acetic acid (10 mL), and concentrated sulfuric acid (0.4 mL) were added to a 100 mL round-bottom flask equipped with a reflux condenser and stirred for 60 min at 100 °C in a constant temperature oil bath. The mixture was poured into a beaker containing 50 g of crushed ice, and stirred continuously until a solid was generated. The resulting solid was filtered, then washed with distilled water until the litmus paper was neutral. Finally, the target compound 4′-methylflavone was purified via silica gel column chromatography (petroleum ether–ethyl acetate = 30:1).

#### 3.2.2. Characterization of 4′-Methyflavanone

For 4′-Methyflavanone, the yield was 81%; it was a white solid, with the following characteristics: mp: 116 °C; ^1^H-NMR (CDCl_3_) δ 8.22 (dd, J = 7.9, 1.5 Hz, 1H), 7.81 (d, J = 8.2 Hz, 2H), 7.71–7.64 (m, 1H), 7.55 (d, J = 8.3 Hz, 1H), 7.40 (t, J = 7.5 Hz, 1H), 7.31 (d, J = 8.1 Hz, 2H), 6.80 (s, 1H), 2.42 (s, 3H); ^13^C-NMR (CDCl_3_) δ 178.48, 163.65, 156.23, 142.29, 133.69, 129.77, 128.90, 126.23, 125.66, 125.15, 123.93, 118.06, 106.91, 21.54; HRMS (ESI-MS) m/z calcd. for C_16_H_13_O_2_ [M + H]+ was 237.09101, and was found to be 237.09109.

### 3.3. Preparation for UV–Vis Spectra, PL Spectra, and SEM Measurements

#### 3.3.1. Preparatory Work before UV–Vis Spectra and PL Spectra Measurements

The flavonoids 1–6 (0.50, 0.53, 0.65, 0.59, 0.72, and 0.72 mg) were weighed accurately with an electronic balance, then dissolved in methanol solvent, mixed well, and prepared as a mother liquor with a final concentration of 2.07 × 10^−4^ M. Then, the mother liquor was diluted 10 times with CH_3_OH/H_2_O mixed solutions of different water contents to obtain solutions to be tested with water contents of 0%, 10%, 20%, 30%, 40%, 50%, 60%, 70%, 80%, and 90%, and the concentration of the final solution was 2.07 × 10^−5^ M. After being sonicated for 15 min, UV–vis absorption spectra and PL spectra compounds **1**–**6** were measured immediately at room temperature with the excitation wavelengths 308, 326, 328, 332, 354, and 340 nm, respectively. The preparation method of compounds **1**–**3** for fluorescence lifetime and fluorescence quantum yield (ϕF) measurements was the same as described above.

#### 3.3.2. Preparatory Work before SEM Measurements

All sample solutions were configured in the same way as described in Section 3.3.1. Compounds **1**–**3** were dissolved in mixed solutions of CH_3_OH/H_2_O with 50%, 60%, and 90% water content, respectively. The abovementioned mixed solutions were sonicated for 15 min at room temperature and then carefully added dropwise to the silicon wafers, separately. After the solvent on the silicon wafers evaporated at room temperature for 24 h, scanning electron microscopy (SEM) imaging was performed.

### 3.4. Preparatory Work before Ethylene Glycol (EG) Measurements

Compounds **1**–**3** were dissolved in a methanol solution, respectively, and mixed well with a final concentration of 2.07 × 10^−4^ M. Then, the solutions were configured as a methanol/EG mixture with EG contents of 0%, 10%, 20%, 30%, 40%, and 50%, respectively. The concentration of the final mixed solution was kept at 2.07 × 10^−5^ M. The PL spectra of compounds **1**–**3** with different EG fractions were measured immediately after sonication at room temperature. The excitation wavelengths of compounds **1**–**3** were 308, 326, and 328 nm, respectively.

### 3.5. Cell Culture

A549 cells were cultured in DMEM medium supplemented with 10% (*v/v*) fetal bovine serum (FBS) and 1% (*v/v*) antibiotics (penicillin, streptomycin) at 37 °C in a humidified atmosphere with 5% (*v/v*) CO_2_. Additionally, the cells were grown as monolayers against the wall.

### 3.6. Cell Viability Assay

The viability of A549 cells was determined via the CCK-8 assay. To perform the experiment, cells were seeded into 96-well plates at a density of 9 × 10^3^ cells per well. The 96-well plates were placed in an incubator for 24 h. After treatment with the prepared solutions of compounds **1**–**3** with different concentrations (1, 5, 10, 15 µM) for 24 h in the incubator, the CCK-8 solution was added to each well. The incubation of the cells was continued for 1.5 h. The absorbance of plates was then monitored using a microplate reader (Molecular Devices) at 450 nm wavelength.

### 3.7. In Vitro Imaging of A549 Cells

The potential cell imaging capability of compounds **1**–**3** and their cellular uptake behavior were investigated via confocal laser scanning microscopy (CLSM, Olympus, FV3000). The study was performed on A549 cells. A549 cells were seeded in 35 mm cultivation dishes at a cell density of 1 × 10^5^ cells per well and incubated for 24 h. Then, the cells were treated with compounds **1**–**3** (20 µM) for 30 min, respectively. After incubation, cells were washed with PBS three times. Finally, the dishes were observed by FV3000 laser scanning confocal microscopy under a 60-fold oil immersion lens. Subsequently, to confirm the localization of compounds **1**–**3** in A549 cells, the sample solutions of compounds **1**–**3** (20 µM) and Mito Tracker Red (MT) (200 nM) were added into a 2 mL culture medium for treatment, respectively. After 30 min of incubation, we used PBS to wash cells three times. Last, cell imaging was performed using the confocal laser scanning microscope under 60-fold and 100-fold oil immersion lenses.

### 3.8. Real-Time Tracking in Zebrafish

Zebrafish were exposed to solutions of compounds **1**, **2**, and **3** (40 μm), and the compounds were dissolved in 0.1% dimethyl sulfoxide (DMSO). Zebrafish were exposed to DAPI (10 µg/mL) as a control at 48 hpf. After 1 h, zebrafish were washed with PBS three times and observed using the EVOS FL Auto imaging system. Then, the compound solutions were incubated in 48 hpf zebrafish for 1 h and 2 h, respectively, and observed using FV3000 laser scanning confocal microscopy. Finally, the zebrafish were continuously cultured in an incubator at 28.5 °C and observed 1 h and 2 h after immersion administration, respectively, at 168 hpf using the EVOS FL Auto imaging system and an image size of 1024 × 1024 pixels. All zebrafish images of Z-stack were taken using the Fiji Java 1.8.0_322 software (National Institutes of Health, Bethesda, MD, USA) [48]. The three-dimensional reconstruction of the whole-body in 168 hpf zebrafish was performed by using the IMARIS V9.0.1 software (Bitplane, Belfast, UK) according to the manufacturer’s instructions.

## 4. Conclusions

In this paper, based on our previous studies on AIEE compounds, we studied six flavonoids and confirmed the significant AIEE properties of compounds **1**–**3** using PL spectroscopy, UV–vis absorption spectra, fluorescence photographs, and fluorescence quantum yields. We confirmed that the main reason for the AIEE properties of compounds **1**–**3** may be their restricted intramolecular rotation (RIR). Furthermore, through a series of cellular experiments, we found that they have low cytotoxicity, good cytocompatibility, and the ability to specifically label mitochondria, suggesting that they have potential applications in the development of mitochondrial imaging and multifunctional probes. We investigated the fluorescence imaging of compounds **1**–**3** in 168 hpf zebrafish and found that they were distributed in the liver and intestine of fully-developed zebrafish. We exploited the fact that compounds have different distributions in zebrafish by cycling for different time periods (between uptake and utilization in the tissue), which is very enlightening for the visualization of pharmacokinetic processes in vivo, tracking the uptake and distribution of drugs, and providing real-time feedback about therapeutic effects. These advances promise a new era of precision medicine, through discovering a series of AIEE compounds which can help us to better understand the correlates between mitochondrial energy metabolism and the liver.

## Figures and Tables

**Table 1:** Fluorescence quantum yield of compounds **1**–**3** in CH_3_OH/H_2_O mixed solution with various water fractions.

Compound	Solvent	Quantum Yield (ϕF)
**1**	CH_3_OH	0.02
	CH_3_OH/H_2_O (5:5)	0.16
	CH_3_OH/H_2_O (1:9)	0.29
**2**	CH_3_OH	0.06
	CH_3_OH/H_2_O (5:5)	0.30
	CH_3_OH/H_2_O (1:9)	0.17
**3**	CH_3_OH	0.23
	CH_3_OH/H_2_O (4:6)	0.68
	CH_3_OH/H_2_O (1:9)	0.51

**Table 2:** Corresponding R values of compounds **1**–**3**.

Compound	Corresponding R Values (Pearson Correlation Coefficient)
**1**	0.86
**2**	0.87
**3**	0.85

## Data Availability

Not applicable.

## Supplementary Materials

The supporting information can be downloaded at https://www.mdpi.com/article/10.3390/ijms241210183/s1.
