# Supplementary material for: AIEE-Active Flavones as a Promising Tool for the Real-Time Tracking of Uptake and Distribution in Live Zebrafish
Source: Int J Mol Sci. 2023 Jun 15;24(12):10183. doi: 10.3390/ijms241210183 (PMC10299116; doi:10.3390/ijms241210183)
Supplement: Supplementary file 1 [file ijms-24-10183-s001.zip › ijms-2419621-supplementary.pdf]

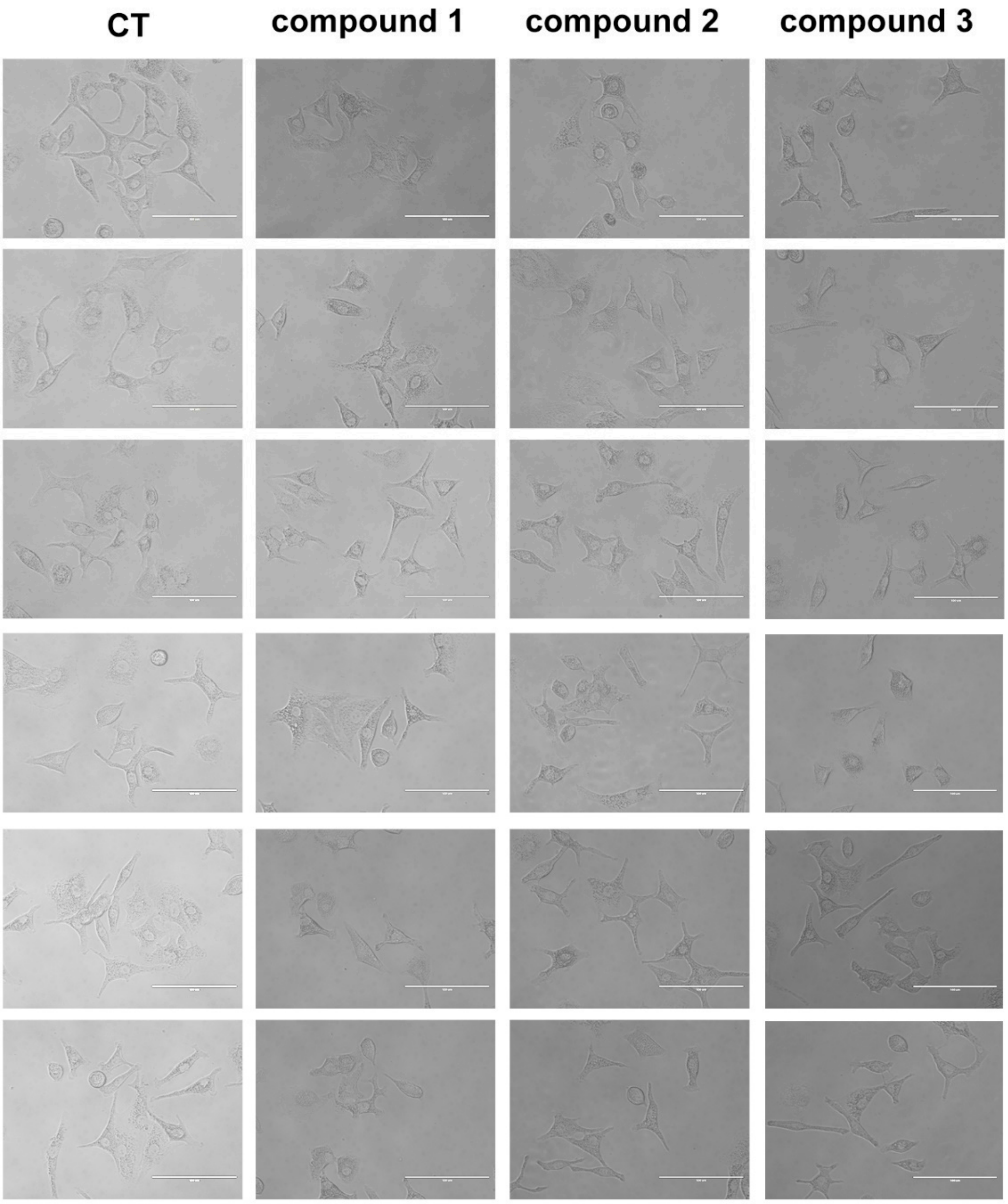

Figure S1. The morphological images of A549 cells. Brightfield images at multiple fields of view indicate that the compounds have no effect on cell morphology. All images were performed under a 40-fold lens. Scale bars, 100 μm.

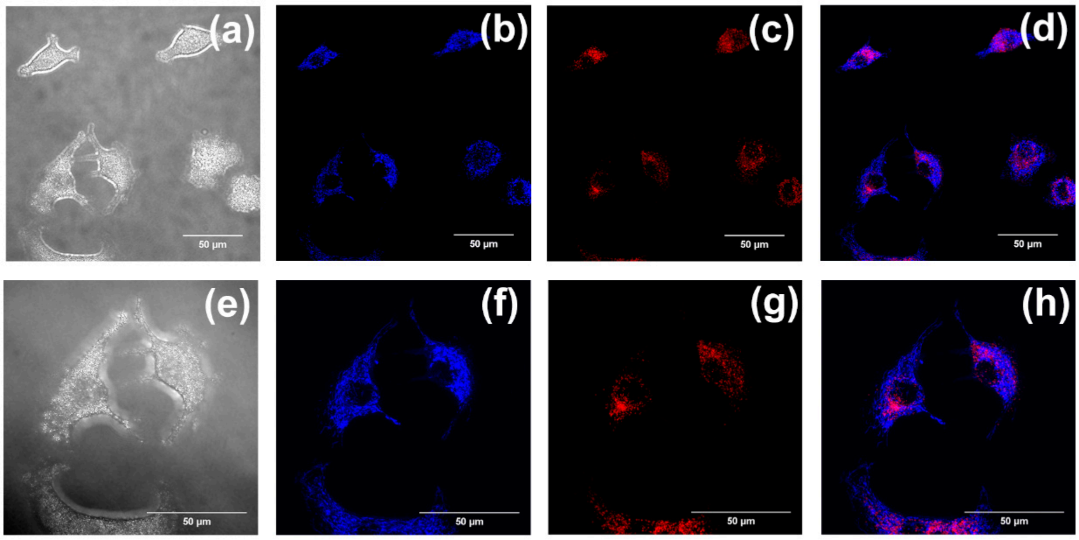

Figure S2. (**a**,**e**) Brightfield pictures of A549 cells. Co-localized images of A549 cells incubated with compound **1** (20 µM) (**b**,**f**) and Lyso-Tracker Red (200 nM) (**c**,**g**) for 30 min. (**d**,**h**) The merged images co-cultured with **1**. The excitation wavelength of compound **1** was 405 nm. The excitation wavelength of Lyso-Tracker Red was 577 nm. (**a**–**d**) were performed on the confocal laser scanning microscope under a 60-fold, and (**e**–**h**) were performed under a 100-fold oil immersion lens. Scale bars, 50 µm.

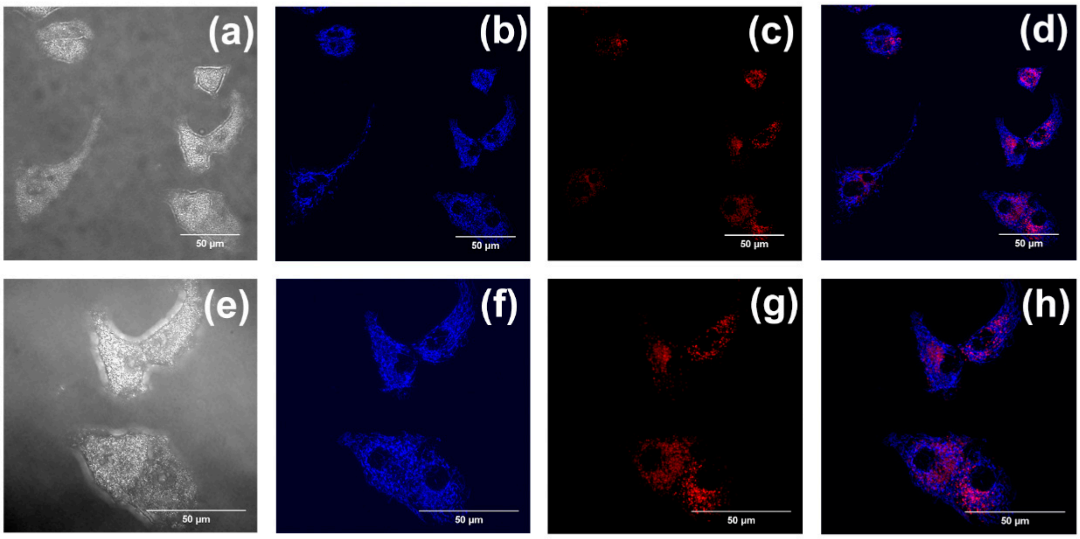

Figure S3. (**a**,**e**) Brightfield pictures of A549 cells. Co-localized images of A549 cells incubated with compound **2** (20 µM) (**b**,**f**) and Lyso-Tracker Red (200 nM) (**c**,**g**) for 30 min. (**d**,**h**) The merged images co-cultured with **2**. The excitation wavelength of compound **2** was 405 nm. The excitation wavelength of Lyso-Tracker Red was 577 nm. (**a**–**d**) were performed on the confocal laser scanning microscope under a 60-fold, and (**e**–**h**) were performed under a 100-fold oil immersion lens. Scale bars, 50 µm.

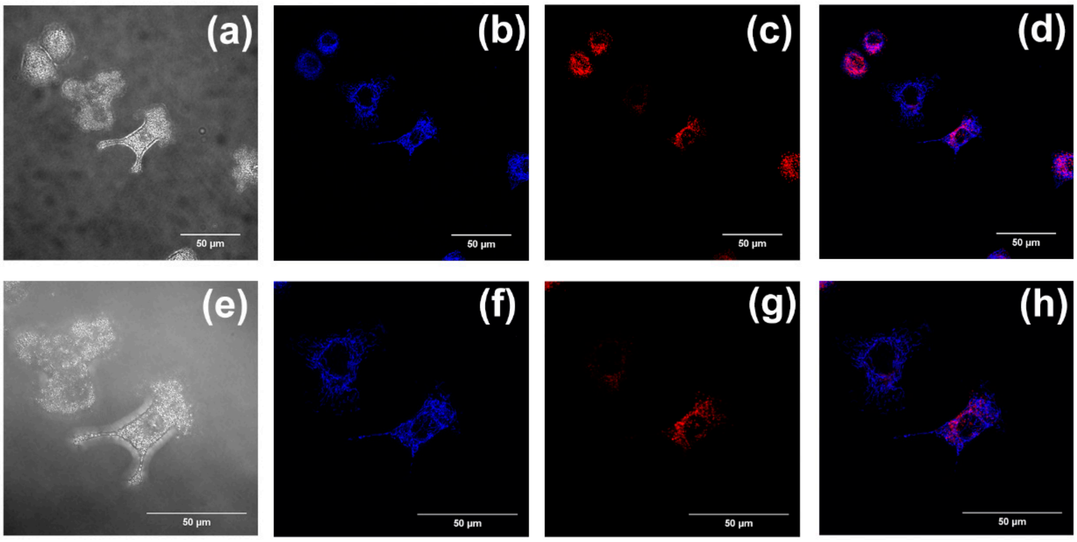

Figure S4. (**a**,**e**) Brightfield pictures of A549 cells. Co-localized images of A549 cells incubated with compound **3** (20 μM) (**b**,**f**) and Lyso-Tracker Red (200 nM) (**c**,**g**) for 30 min. (**d**,**h**) The merged images co-cultured with **3**. The excitation wavelength of compound **3** was 405 nm. The excitation wavelength of Lyso-Tracker Red was 577 nm. (**a–d**) were performed on the confocal laser scanning microscope under a 60-fold, and (**e–h**) were performed under a 100-fold oil immersion lens. Scale bars, 50 μm.

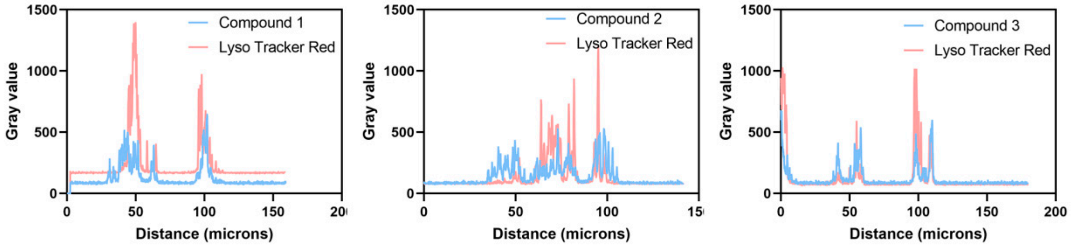

Figure S5. The Plot Profile of compounds **1**–**3** and Lyso Tracker Red.

Table S1. Corresponding R values of compounds **1**–**3**.

Compound	Corresponding R Values (Pearson Correlation Coefficient)
**1**	0.37
**2**	0.53
**3**	0.56

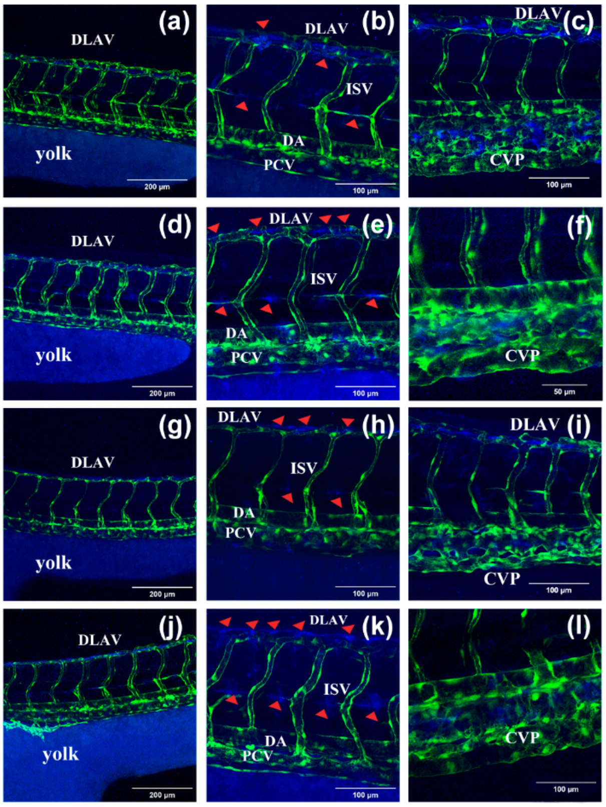

Figure S6. (**a**,**b**) and (**g**,**h**) Fluorescence images of Tg(fli1a:EGFP) after incubation with compound **1** (40 µM) and compound **3** (40 µM) for 1 h in 48 hpf larve, respectively. In (**b**), most of the compound adhere to the surface of the blood vessels, and a few enter the blood vessels. (**d**,**e**) and (**j**,**k**) Fluorescence images of Tg(fli1a:EGFP) after incubation with compound **1** (40 µM) and compound **3** (40 µM) for 2 h at 48 hpf, respectively. Arrowheads in (**b**,**e**) and (**h**,**k**) indicates the compound **1** and compound **3** adhere to the surface of the blood vessels, respectively. In (**e**,**k**), the amount of compounds absorbed into the blood vessels is greatly increased. The compounds are indicated by triangles and numbered in red in (**b**,**e**,**h**,**k**). (**c**,**f**) and (**i**,**l**) The compound **1** and compound **3** entered the CVP. Scale bars, 200 µm (**a**,**d**,**g**,**j**), 100 µm (**b**,**c**,**e**,**h**,**i**,**k**,**l**), 50 µm (**f**).

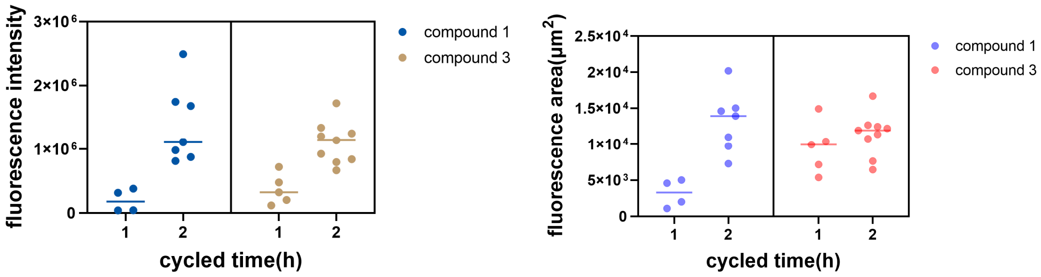

Figure S7. Fluorescence intensity of compounds **1** and **3** cycled for 1 h and 2 h, respectively and fluorescence area of compounds **1** and **3** cycled for 1 h and 2 h, respectively. The number of dots in the Figure S7 corresponds to the red triangles in the Figure S6.

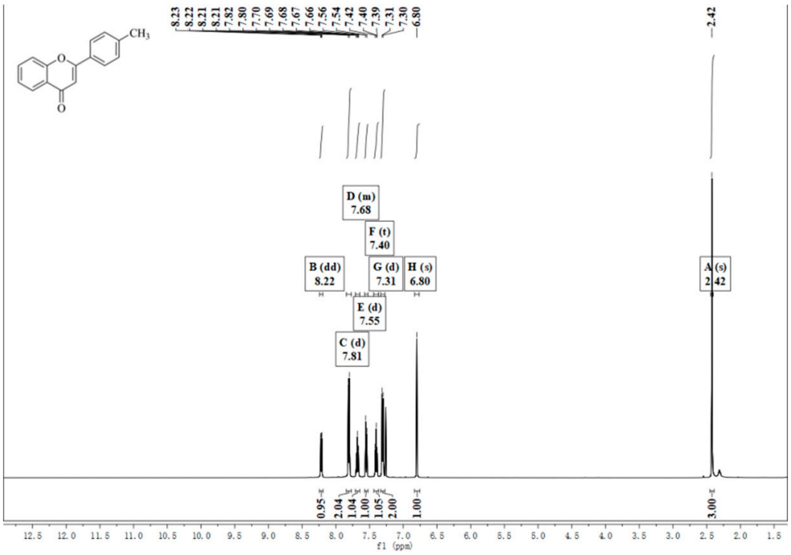

Figure S8. ^{1}H-NMR of compound **1**.

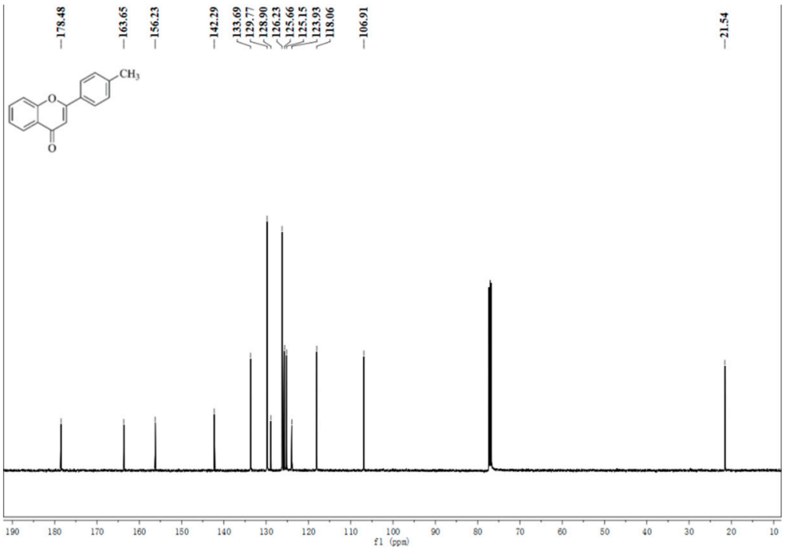

Figure S9. ^{13}C-NMR of compound **1**.

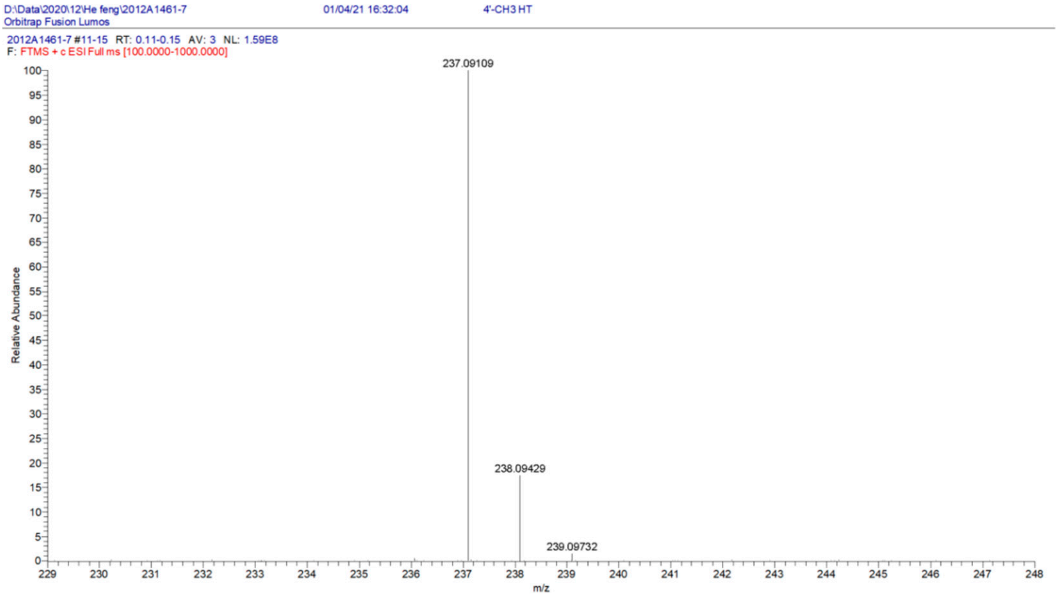

SPECTRUM - simulation :

m/z	Theo. Mass	Delta (ppm)	RDB equiv.	Composition
237.09109	237.09101	0.35	10.5	$C_{16}H_{13}O_2$

Limits:

1) Charge: +1
2) Nitrogen-Rule: Do not use
3) Mass tolerance: 5 ppm
4) Elements in use: ^{12}C (0~30), ^{1}H (0~60), ^{16}O (0~10), ^{14}N (0~5)

Figure S10. High-Resolution Mass Spectrometry (HRMS) of compound **1**.

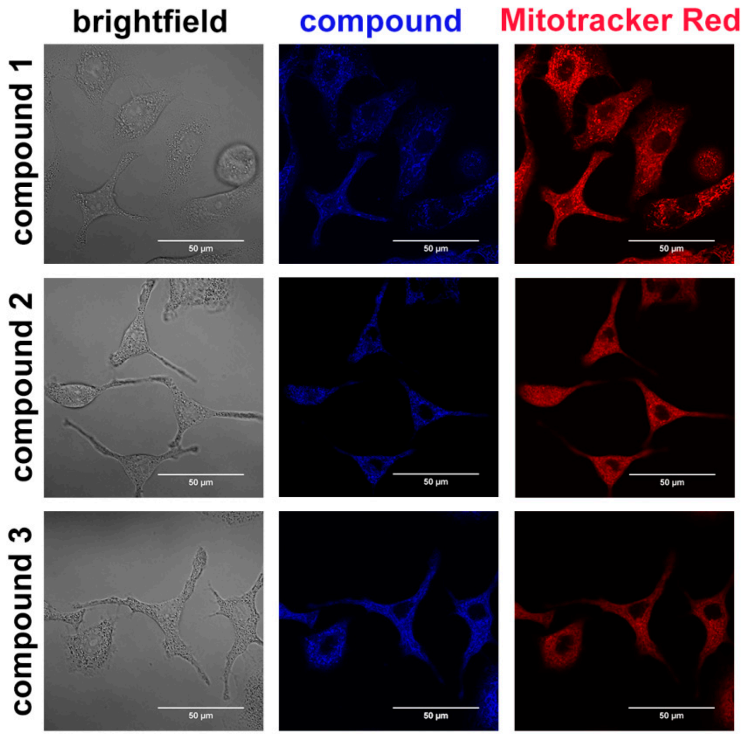

Figure S11. The two-dimension co-localized images of A549 cells incubated with compounds **1**–**3** (20 μM) and Mito Tracker Red (200 nM) for 30 min. Scale bars, 50 μm.

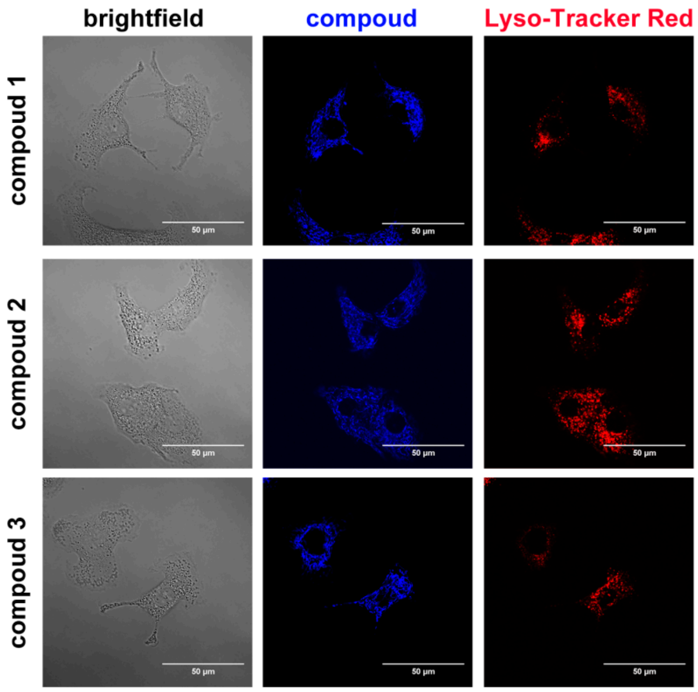

Figure S12. The two-dimension co-localized images of A549 cells incubated with compounds **1**–**3** (20 μM) and Lyso-Tracker Red (200 nM) for 30 min. Scale bars, 50 μm.
